# Aesthetic satisfaction of patients following plastic surgery of nail folds combined with conservative nail plate reconstruction for ingrown toenails

**DOI:** 10.3389/fmed.2025.1732863

**Published:** 2026-01-20

**Authors:** Tomasz Trochanowski, Aleksandra Dańczak Pazdrowska, Ewa Baum

**Affiliations:** 1 Department of Social Sciences and the Humanities, Poznan University of Medical Sciences, Poznan, Poland; 2 Zrodlana Medical Centre, Zielona Gora, Poland; 3 Top Medical Clinic, Isleworth, London, United Kingdom; 4 Department of Dermatology, Poznan University of Medical Sciences, Poznan, Poland

**Keywords:** aesthetic podiatry, conservative nail plate reconstruction, ingrown toenail, nail fold plastic surgery, quality of life

## Abstract

**Introduction:**

Management of ingrown toenails encompasses a wide range of methods, from conservative to surgical approaches, which vary in terms of final aesthetic outcomes and overall patient satisfaction.

**Aim:**

This study aimed to assess patient aesthetic satisfaction following surgical correction of nail folds preceded by conservative nail plate reconstruction.

**Materials and methods:**

A total of 550 procedures were performed in 340 patients, involving plastic surgery of the nail folds combined with conservative nail plate reconstruction. Patients evaluated their satisfaction with the aesthetic and functional outcomes of the operated toes.

**Results:**

The final aesthetic appearance of the treated toes was rated as good (34.4%) or very good (59.4%) by approximately 95% of patients. Significant relationships were observed between the final aesthetic outcome and post-treatment effectiveness (*p* = 0.003), severity of ingrown toenail prior to treatment, need for additional cosmetic and conservative procedures (*p* = 0.008), patient age (*p* < 0.0001), and type of occupation (*p* = 0.0016).

**Conclusion:**

Plastic surgery of the nail folds combined with conservative nail plate reconstruction achieved favorable final aesthetic outcomes for the toes and nail plates, contributing to high patient satisfaction.

## 1 Introduction

From the very beginning, humans have paid attention to the appearance and aesthetics of their bodies, which can be negatively affected by illness or its treatment (1, 2). A lack of a normal, physiological body appearance—both before and after treatment—has often been associated with a reduction in quality of life and may even contribute to various psychological and social disorders (1, 3, 4).

An example of a condition that affects both medical and aesthetic aspects is an ingrown toenail (5). Ingrown toenails most commonly affect the hallux and result from a disruption in the physiological balance between the nail plate and the surrounding nail folds (6, 7). The continuity of the nail fold is interrupted by a damaged nail or its fragment, leading to an inflammatory process if not addressed in the early stages (5, 8, 9).

This condition can be treated using a variety of conservative and surgical methods, often in combination (10–12). Conservative management is typically the first-line treatment, especially in the early stages of the disease, and is usually guided by 3- or 4-point clinical scoring systems (13–15). Such treatment is often effective, relieving pain and allowing patients to return to normal daily activities (16).

Although conservative treatment may initially be successful, non-surgical methods may prove ineffective in the long term, with recurrence of pain and inflammation (12, 17). In such cases, the same conservative approach may be continued, or an alternative non-surgical method may be considered (12). Surgical intervention is usually regarded as a second-line treatment, but may be used as first-line therapy in advanced cases of toenail ingrowth (8, 12).

In general, surgical treatments for ingrown toenails can be categorized into two types: procedures involving removal of the nail plate (or part of it), and those that preserve the nail plate while correcting the nail folds (18–20). Currently, there is no universally accepted surgical method for the treatment of ingrown toenails (6, 13, 21).

All available methods, both conservative and surgical, differ in their approach, effectiveness in symptom relief, and in the final aesthetic outcome of the treated toe and nail plate (5, 22–26). In many cases, patients no longer experience the bothersome symptoms of an ingrown toenail but remain dissatisfied with the appearance of the toe and nail plate, which can negatively impact their quality of life (8, 27, 28).

Therefore, any treatment for ingrown toenails should consider its effect on the aesthetic outcome of the toe and nail plate, aiming to ensure full patient satisfaction (5, 25, 26). The aim of this study was to assess patient satisfaction following surgical correction of the nail folds, preceded by conservative reconstruction of the nail plate.

## 2 Materials and methods

Between 2018 and 2021, a group of 340 patients (aged 10–64 years) underwent surgical correction of the nail folds, preceded by conservative reconstruction of the nail plate. A total of 550 procedures were performed: 130 patients underwent the procedure on one hallux, and 210 patients on both halluces. The indication for the procedure in all cases was an ingrown toenail. Patients participating in the study were selected consecutively. No specific inclusion or exclusion criteria were applied beyond the standard clinical qualifications required for the procedure.

Symptom severity was assessed by a physician using a 3-point scale according to the Heifetz classification, where 3 indicated the most severe condition. In patients who received treatment for both halluces, the severity of ingrown toenails was assessed independently for each toe. Notably, in all of these cases, the severity was identical bilaterally.

Patients underwent follow-up at least 6 months after surgery. The median follow-up duration was 6 months (range: 6–12 months), during which they were asked to evaluate both the aesthetic outcome and the overall effectiveness of the procedure. All assessments were conducted after complete wound healing and nail regrowth, ensuring consistency in outcome evaluation. Responses were collected using a four-point scale: *poor, satisfactory, good,* and *very good.* Effectiveness was defined as a broader concept than aesthetics alone, encompassing the absence of ingrown toenail recurrence and the patient’s overall satisfaction with the therapy. The aesthetic satisfaction scale used in this study was developed specifically for this project and was not adapted from any existing patient-reported outcome measure (PROM). Patients completed the self-assessment independently to minimize potential bias and without any influence from the treating physician.

Following the main treatment, a subgroup of 105 patients underwent additional procedures aimed at enhancing the final aesthetic appearance of the operated toe and nail plate. This adjunct therapy included both cosmetic and conservative methods, such as placing small wisps of cotton or other material under the edge of the ingrown nail or applying a nail brace. It should be noted that these additional procedures did not determine the successful resolution of ingrown toenail symptoms in the treated patients but rather supported tissue and nail regeneration, thereby improving the final aesthetic result.

The study was approved by the Bioethics Committee of the University of Zielona Gora (no. KB-UZ/4/2021).

Characteristics of patients included in the study are illustrated in Table 1.

**Table 1:** Characteristics of patients included in the study.

Characteristic	n (%)
Gender	
Male	235 (69%)
Female	105 (31%)
Type of occupation	
Office/school	230 (67.6%)
Manual work	95 (28%)
Mixed	15 (4,4%)
Number of operated toes	
One hallux	130 (38.2%)
Both halluces	210 (61.8%)
Additional procedures performed	105 (31%)

### 2.1 Surgery of the nail folds preceded by conservative reconstruction of the nail plate

**Figure 1:**
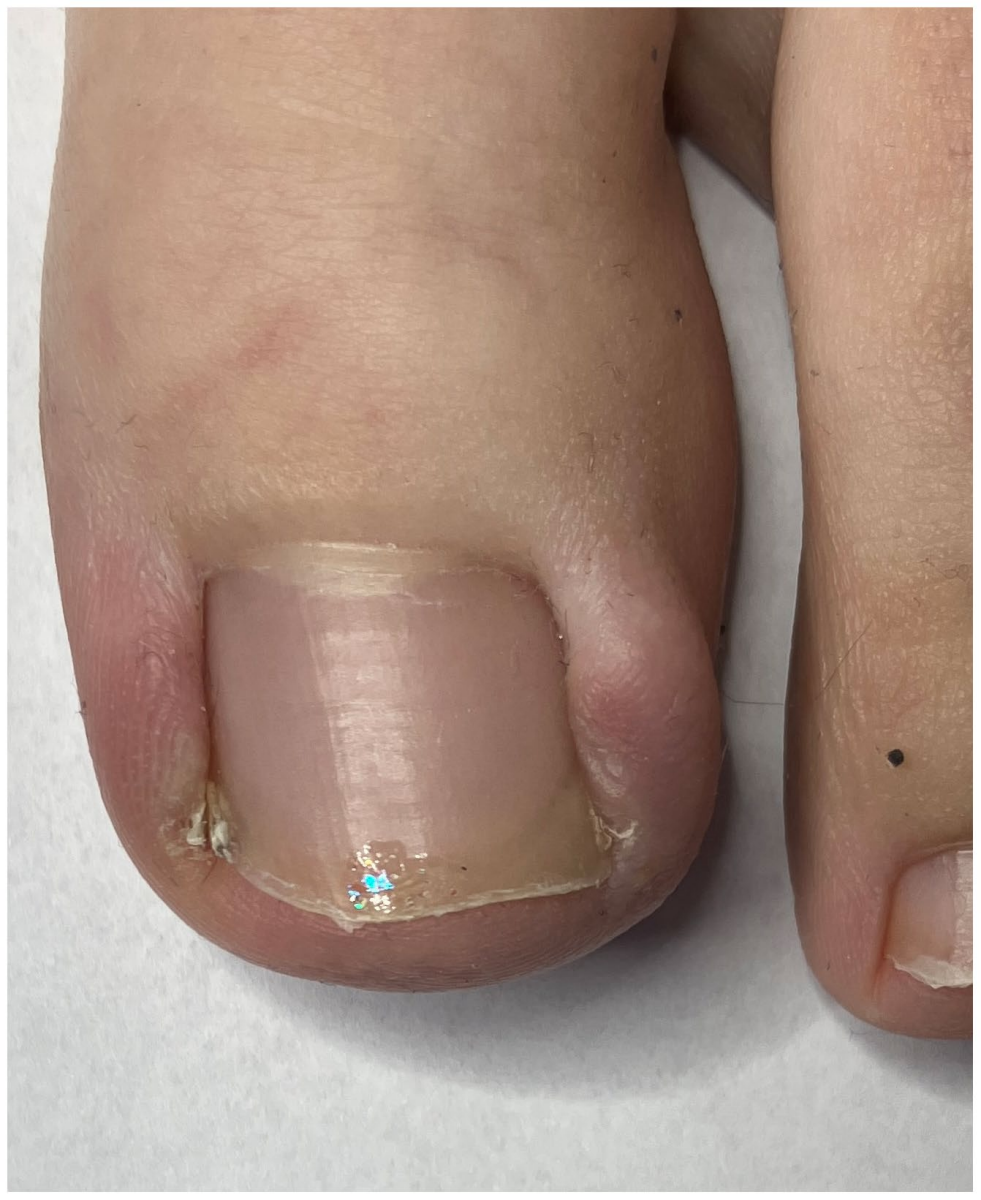
Before the procedure.

**Figure 2:**
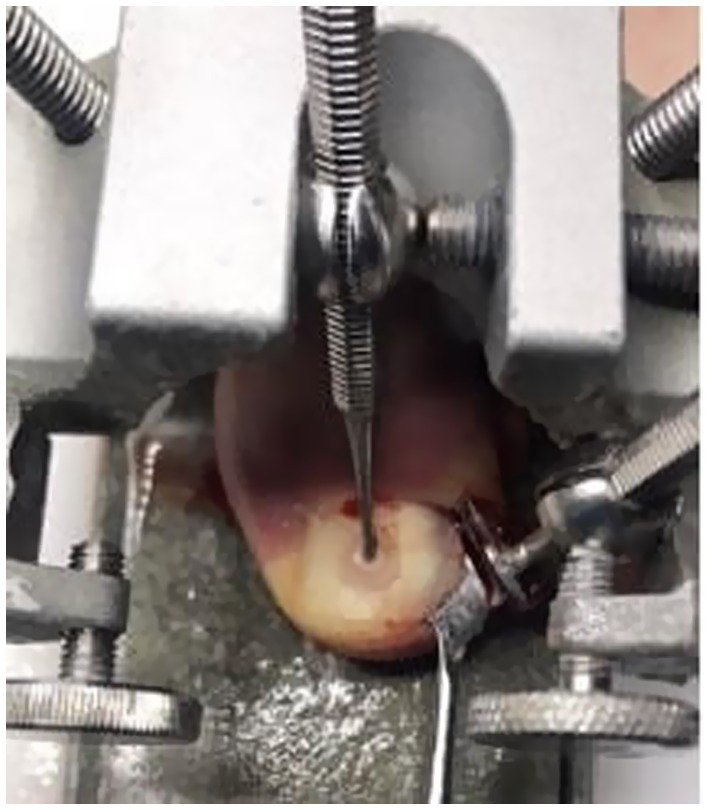
Conservative nail plate reconstruction in Arkada’s cube.

**Figure 3:**
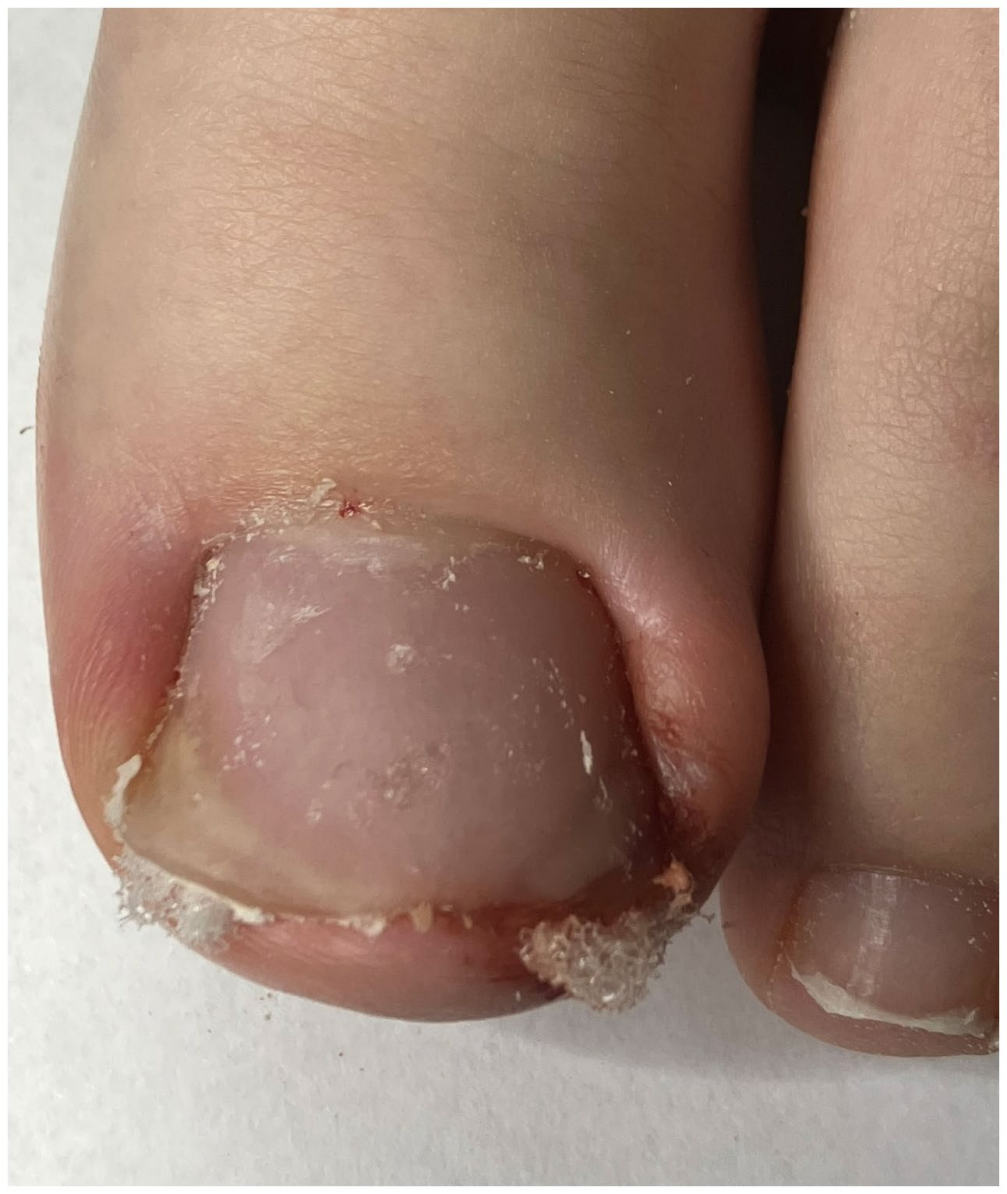
Immediately after conservative nail plate reconstruction.

**Figure 4:**
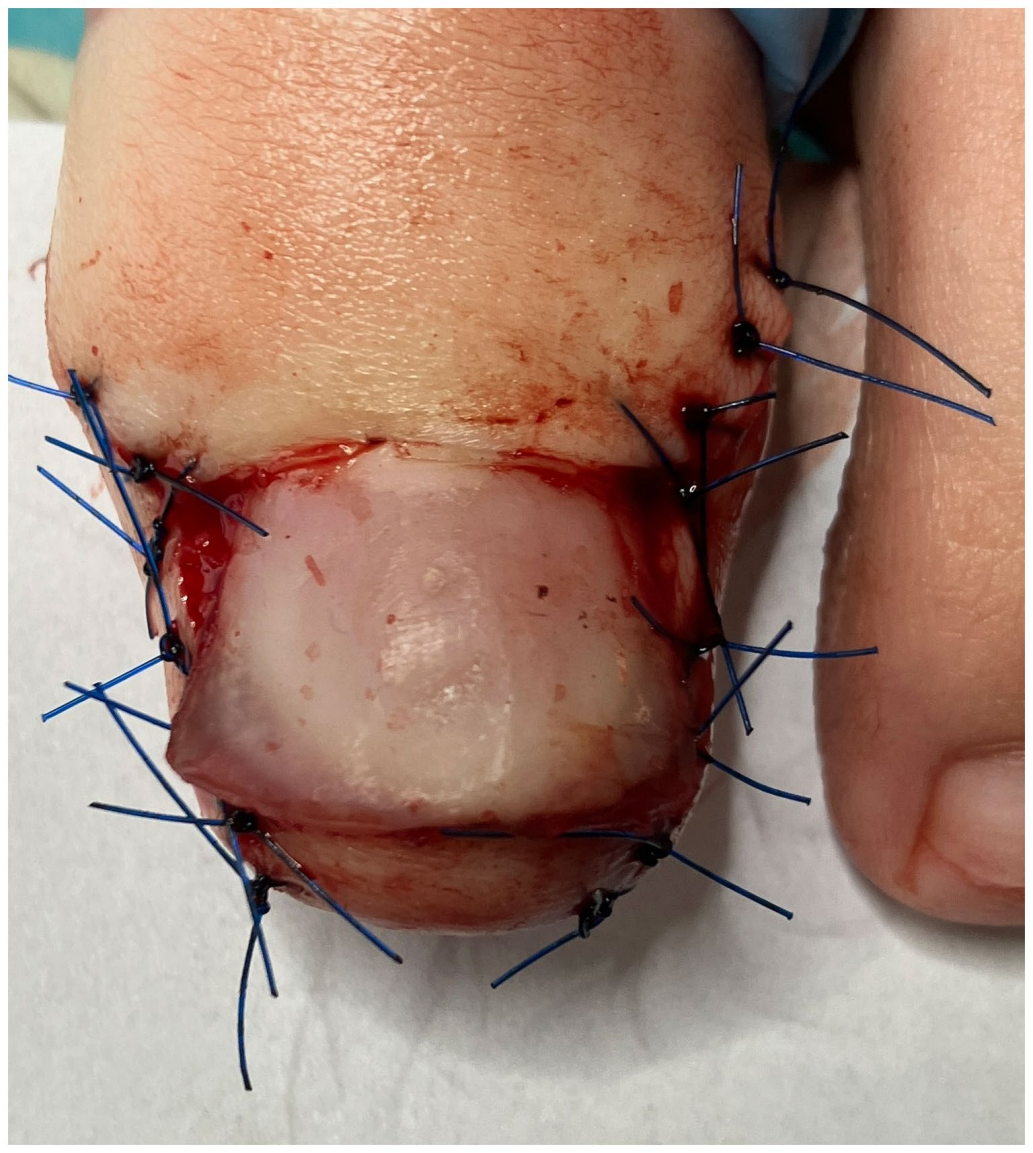
Immediately after plastic surgery of the nail folds.

**Figure 5:**
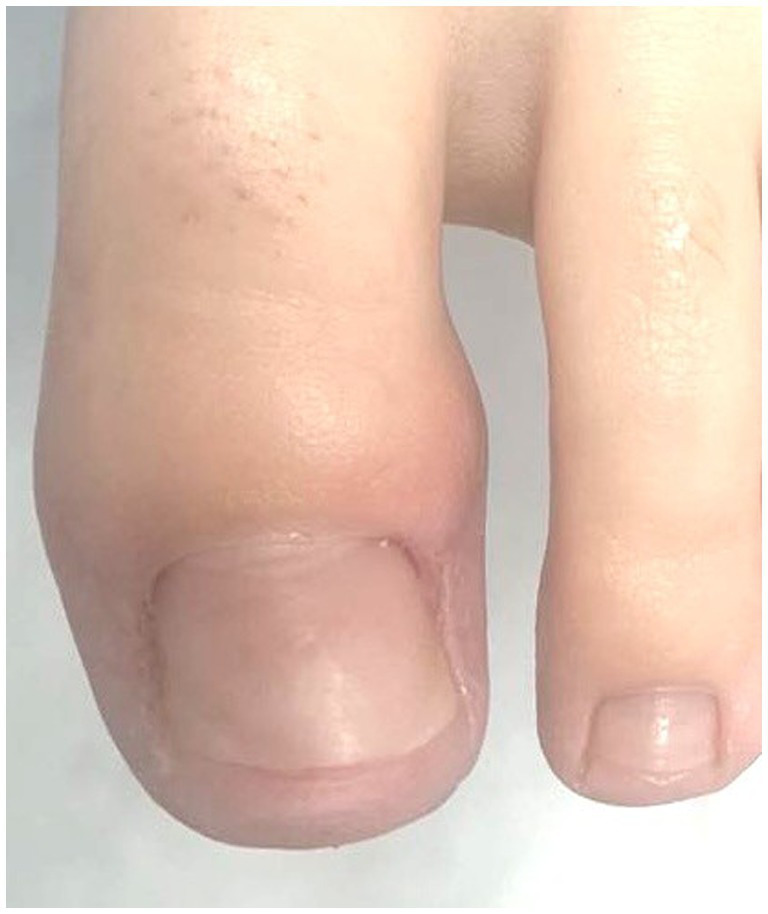
Final appearance.

In this study, each surgical correction of the nail folds was preceded by conservative reconstruction of the nail plate, performed using Arkada’s method. This podiatric, non-surgical treatment aims to restore the nail plate, which is often deformed or incomplete due to accompanying inflammation or previous (most often surgical) interventions. The procedure is carried out with a specialized device called Arkada’s cube, which enables non-invasive repositioning of the hypertrophic nail folds, thereby exposing the nail plate and allowing its defects to be corrected with materials such as acrylic or composite (10, 12, 29). Repairing the nail plate and covering its surface with these materials helps restore its physiological shape and hardness (12, 29). As a result, the reconstructed nail plate—elevated and reinforced with acrylic or composite material—can grow properly, while the surrounding folds are no longer irritated by the previously damaged plate. Conservative nail reconstruction is typically performed immediately before the next stage of treatment - plastic surgery of the nail folds. The procedure is carried out under local anesthesia, and sutures are removed approximately 2 weeks later. Recommended postoperative care includes natural, physiological walking and gentle pressure on the operated toe to promote proper healing. The procedure used in the study is presented in Figures 1–5.

### 2.2 Statistical analysis

All statistical analyses were performed using Dell Statistica (version 13; Dell Inc., 2016). Normality of variable distributions was assessed using the Shapiro–Wilk test. Quantitative variables that did not follow a normal distribution or were measured on an ordinal scale were presented as the median (min–max) or median (Q25–Q75), while categorical variables were expressed as n (%). Statistical significance was determined at a two-tailed significance level of *α* = 0.05. For quantitative variables with normal distributions, parametric tests were applied, including analysis of variance (ANOVA) for comparisons between groups. For variables without normal distributions or those measured on an ordinal scale, non-parametric tests were used, such as the Mann–Whitney U-test, the Wilcoxon signed-rank test, and Spearman’s rank correlation coefficient. Associations between qualitative variables were examined using the chi-square test with Yates’ correction, the standard chi-square test, or Fisher’s exact test, as appropriate. Multiple comparisons were performed using the Bonferroni correction; therefore, *p*-values reported reflect adjusted significance thresholds.

## 3 Results

According to the clinical scoring system, the symptom severity of ingrown toenails in the studied patients was as follows: stage 1–5 patients, stage 2–131 patients, and stage 3–204 patients. The symptom severity is illustrated in Table 2.

**Table 2:** Symptom severity of ingrown toenails in the studied patients (Heifetz classification).

Symptom severity	n (%)
Stage 1	5 (1.5%)
Stage 2	131 (38.5%)
Stage 3	204 (60%)

Regarding post-treatment effectiveness, the majority of patients rated the outcome as good (*n* = 106; 31.2%) or very good (*n* = 225; 66.2%). Only a small proportion of patients (*n* = 9; 2.7%) assessed the results as fair or poor.

Similarly, almost 95% of patients described the final aesthetic appearance of their toes as good (34.4%) or very good (59.4%). Lower ratings were reported by a minority of patients, with 5% (*n* = 17) assessing the results as fair and 1.2% (*n* = 4) as poor.

**Figure 6:**
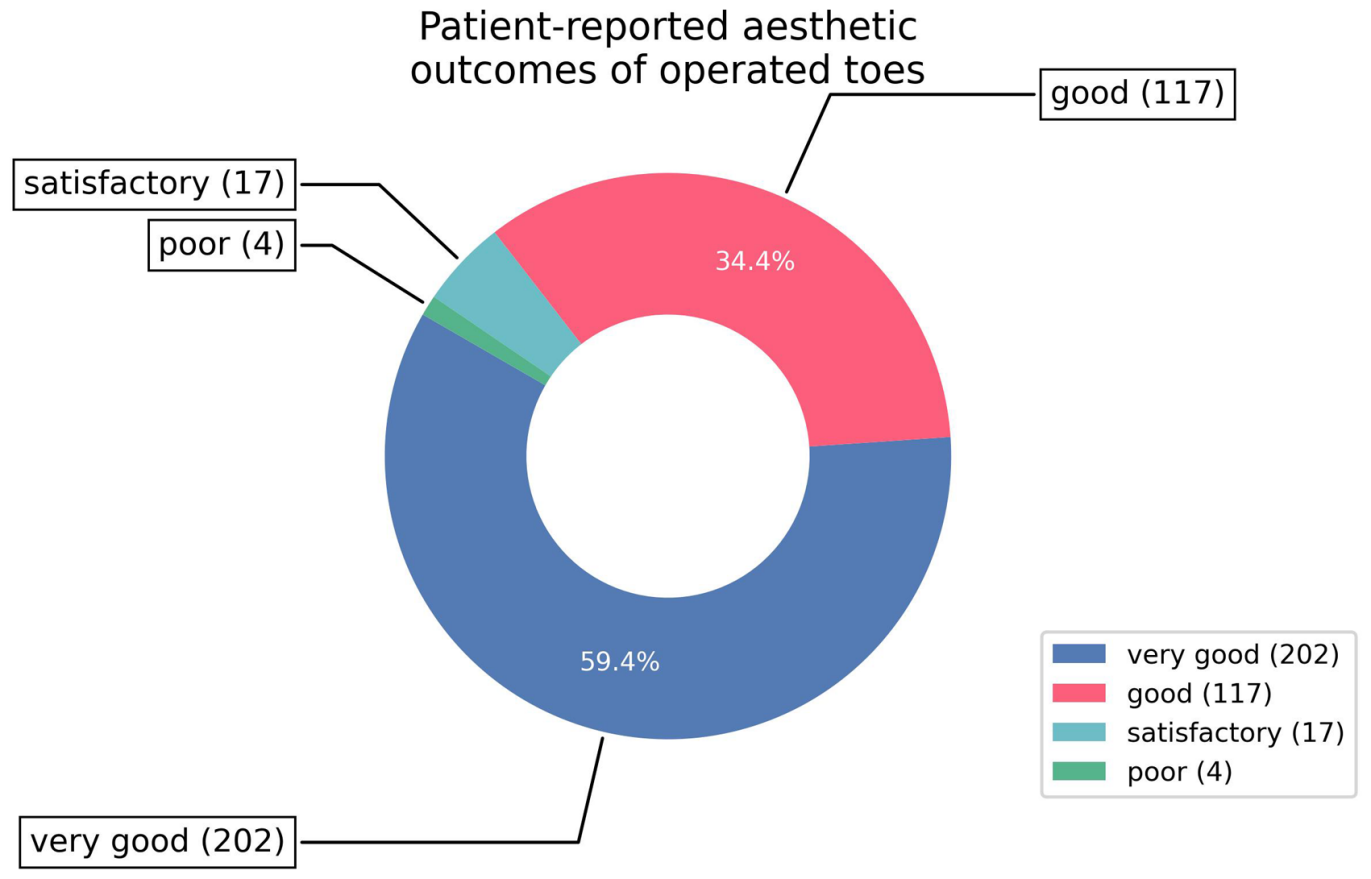
Patient-reported aesthetic outcomes of operated toes.

Post-treatment effectiveness and aesthetic outcomes are illustrated in Table 3 and Figure 6.

**Table 3:** Post-treatment effectiveness and aesthetic outcomes: distribution of patients by number and percentage (n, %).

Assessment category	Poor	Fair	Good	Very good
Post-treatment effectiveness	3 (0.9%)	6 (1.8%)	106 (31.2%)	225 (66.2%)
Aesthetic outcome	4 (1.2%)	17 (5%)	117 (34.4%)	202 (59.4%)

A significant association was found between the baseline severity of ingrown toenails and patients’ final aesthetic evaluation (Fisher–Freeman–Halton test, *p* = 0.003; Fisher’s exact test, *p* = 0.0007; Bonferroni-adjusted *α* = 0.0028; chi-square with Yates’ correction, *p* < 0.0001). *Post hoc* analysis showed that patients with stage 3 severity were significantly more likely to report good or very good aesthetic outcomes compared with those with stage 1 severity.

Spearman’s rank correlation analysis demonstrated a strong, statistically significant positive correlation between final aesthetic outcomes and post-treatment effectiveness (*r*s = 0.74, *p* < 0.0001). This indicates that higher aesthetic ratings were associated with higher perceived effectiveness of the procedure.

Another significant association was observed regarding the need for additional cosmetic or conservative procedures following plastic surgery on the nail folds (Fisher–Freeman–Halton test, *p* = 0.008; chi-square with Yates’ correction, *p* = 0.0376; chi-square test, *p* = 0.0018). Patients who did not require supplementary procedures were more likely to rate the final aesthetic outcome as very good or good, whereas those who underwent additional interventions more frequently assessed the results as only satisfactory.

The Spearman’s rank correlation coefficient demonstrated a weak but statistically significant association between patient age and the final aesthetic assessment of the toes following plastic surgery of the nail folds (*r*s = −0.276, *p* < 0.0001). This association was inversely proportional, indicating that older patients tended to report lower aesthetic satisfaction compared to younger patients. In contrast, no significant difference was observed between women and men in their perception of the aesthetic outcome (*p* = 0.938).

A further significant association was identified between final aesthetic assessment and the type of work performed by the patients (Fisher–Freeman–Halton test, *p* = 0.0016; chi-square, df = 1, *p* = 0.0006). Patients working in office settings or attending school were more likely to rate the final appearance as very good, whereas individuals engaged in manual labor more often rated the result as good rather than very good.

## 4 Discussion

The present study was primarily focused on assessing patient satisfaction with the treatment of ingrown toenails, with particular emphasis on the final appearance of the toe and nail following plastic surgery of the nail folds, preceded by conservative nail plate reconstruction. In addition to evaluating aesthetic outcomes, patients also assessed the overall post-treatment effectiveness of the procedure. In both domains, good and very good ratings predominated, underscoring the high effectiveness of the applied medical procedure. This highlights that for patients, not only the resolution of symptoms but also the cosmetic outcome of their toes may play an important role in their perception of treatment success. Such findings are consistent with the broader scientific literature, where aesthetic appearance is a key determinant of psychosocial wellbeing (1, 4).

This finding is particularly meaningful given that, in the vast majority of cases, the severity of ingrown toenail symptoms was at least moderate or severe (10, 12). Unlike aesthetics and effectiveness, which were subjectively assessed by patients, the severity of ingrown toenails was determined during study qualification. The observed association between baseline symptom severity and patients’ assessment of final toe aesthetics was somewhat unexpected. This is because treating more severe ingrown toenails, often accompanied by complications from previous unsuccessful treatments, can be technically challenging, requires longer healing, and does not always guarantee a favorable aesthetic outcome (5, 8, 12).

On the other hand, patients presenting with a more advanced disease may report higher satisfaction for at least two reasons. First, in these cases, treatment often restores a more physiological or at least corrected appearance of the toes and nail plates. Second, the procedure frequently marks the conclusion of a prolonged and previously ineffective therapeutic process, which may have been additionally burdened by complications from earlier interventions. These considerations may reflect the fact that patients with the greatest initial impairment experienced the most visible improvement after treatment, whereas patients with milder symptoms might have had less room for noticeable aesthetic changes and therefore assessed outcomes more critically. This interpretation is further supported by the high post-treatment effectiveness scores and the significant correlation between perceived effectiveness and final aesthetic outcomes (22, 25). Importantly, the high ratings of overall post-treatment effectiveness, understood as a broad construct, may reflect not only the positive final aesthetic outcome but also other factors, such as rapid recovery and an undisturbed return to daily activities (30, 31).

Referring to the above-mentioned completion of treatment, this primarily means the disappearance of symptoms related to ingrown toenails, such as pain and inflammation, and consequently the inability to classify any severity of the condition. In most studies, the further course of patient recovery after therapy is rarely discussed, with emphasis placed instead on the final cure and the approximate time needed for patients to return to pre-treatment functionality including both normal daily and professional activities (22, 25, 30–34). However, in cases of long-standing severe inflammation or multiple previous surgical procedures, the nail is often temporarily or even permanently damaged. As a result, the time required to regain satisfaction with the appearance of the treated toe and nail may extend for many weeks or even months following the main procedure. Moreover, permanent damage to the nail matrix or the nail plate itself may prevent patients from ever achieving full satisfaction (8, 25, 28, 35). It should therefore be emphasized that the absence of symptoms does not automatically imply complete regeneration of the toe and nail, nor does it guarantee patient satisfaction (4, 26).

Therefore, in some patients, after stitch removal 2 weeks following plastic surgery of the nail folds, additional cosmetic procedures or conservative treatment were required. This was mainly due to incomplete regeneration of the nail plate and delayed restoration of its original appearance, which in turn necessitated further follow-up visits to the podiatry office. The lack of a rapid and satisfactory improvement in the appearance of the toes and nail plates, combined with prolonged convalescence, may have significantly influenced patients’ assessments of the final aesthetic outcome, which was associated with lower satisfaction scores. This observation supports the significant association between these two parameters. It should be emphasized, however, that failure to provide additional therapy would not have prevented recovery; rather, it might have prolonged the healing process, and in some cases, the previously damaged nail plate might never have fully regained its pre-symptom appearance.

The duration of toe and nail plate regeneration was also influenced by patient age; namely, high satisfaction with the final aesthetic appearance, although observed in the majority of patients, showed a significant inverse association with age. The explanation for this finding could be explained by age-related comorbidities, lower tissue elasticity, and degenerative changes in the nails, such as nail involution (36–42). Consequently, it may have prevented patients from achieving the aesthetic outcomes they expected.

The significant association between the final aesthetic outcome of the treated toe and the type of work performed by patients has not been previously reported in the literature on ingrown toenails. Existing studies mainly emphasize that this podiatric condition can impair the ability to work or attend school and may delay the return to professional or educational activities after treatment, thereby affecting overall wellbeing (18, 22, 25, 32, 33). The duration of work absence may also vary depending on the treatment method used. In addition, postoperative restrictions, such as limited walking or reduced weight-bearing on the operated toe during the early recovery phase, can further prolong the return to work (22, 25).

In the case of the comprehensive treatment used in the present study, patients were recommended to walk immediately after the procedure. Generally, early post-treatment mobilization supports efficient healing by ensuring adequate blood supply to the tissues and reducing the risk of swelling in the foot and lower limb. As a result, patients could often return to office work or school almost immediately after surgery, which may also have contributed to their positive assessment of both the effectiveness of the procedure and the final aesthetic outcome of the toe and nail plate (10, 12).

Regarding the type of work performed by the examined patients, office work has a less negative impact on the healing of the operated toe, partly because it does not require the use of restrictive work shoes and allows for the possibility of wearing open or recreational footwear during recovery (43). Moreover, aesthetic appearance may play a greater role for office workers than for manual workers, as open and more elegant shoes are often worn in professional settings, exposing the toes and feet. Consequently, the loss of a physiological appearance of the toes and toenails due to ingrown toenails may pose a greater problem for office workers compared to manual ones, for whom aesthetics are of relevance mainly in recreational contexts rather than in both professional and social settings (1, 2).

The presented study is innovative for several reasons. First, the applied method of treating ingrown toenails, combining conservative (podiatric) therapy with surgical intervention in a single comprehensive approach, is rarely described in the scientific literature (10, 12). When considered individually, both components of the treatment, namely conservative nail plate reconstruction and plastic surgery of the nail folds, are also used less frequently compared to more common approaches (5, 13). In podiatric practice, for instance, braces are more often applied, while in surgical treatment, partial nail excision with or without permanent matrix destruction remains the standard of care (5).

In addition to the rarity of the treatment approach itself, the assessment of patient satisfaction with the aesthetic outcome after ingrown toenail treatment is only sporadically reported. Most studies focus instead on the objective clinical effectiveness of surgical procedures, with particular emphasis on those involving direct intervention on the nail plate (23, 25, 26, 44). Moreover, to our knowledge, no previous publications have examined the associations between the final aesthetic result and factors such as the initial severity of ingrown toenails, the need for additional conservative procedures to achieve full recovery, or the type of work performed by the patients, as presented in this study. Overall, our findings highlight the importance of integrating patient-reported outcomes, particularly aesthetic satisfaction, into the evaluation of treatment strategies for ingrown toenails. A combined consideration of both functional and aesthetic outcomes may support more individualized therapeutic decision-making and ultimately strengthen patient-centered care (45).

The study has several limitations. First, it was conducted at a single center, and all patients were treated by the same physician. Second, the study was not randomized; although participants represented different age groups and social backgrounds, they belonged to a single ethnic group and were predominantly from one country. These two limitations may reduce the generalizability of the findings due to the influence of a single clinician’s expertise and the ethnic homogeneity of the study population. Third, no direct comparisons were made with other methods of treating ingrown toenails, which limits the ability to conclude the relative advantages of the technique used in this study. The absence of a control or comparison group further restricts any inference regarding the superiority of this method; therefore, future comparative or randomized studies are needed. Finally, the study relied on an *ad hoc* survey rather than a standardized questionnaire. While numerous patient-reported outcome measures (PROMs) and quality-of-life questionnaires exist, including some addressing the appearance of treated body parts such as nails, there is no validated tool specifically designed for patients with ingrown toenails (46–51). Given that the main focus of the present study was on aesthetics, the use of an ad hoc instrument was deemed appropriate, although it remains a methodological limitation.

## 5 Conclusion

The plastic surgery of nail folds combined with conservative reconstruction of the nail plate resulted in high patient satisfaction regarding the final aesthetic appearance of the operated toes. Compared with other surgical methods, this approach appears to minimize complications such as nail deformities while maximizing aesthetic and functional outcomes. Significant associations were found between the final aesthetic outcome and the post-treatment effectiveness, the initial severity of ingrown toenails, the need for additional cosmetic and conservative procedures after treatment, patient age, and the type of work.

## Data Availability

The raw data supporting the conclusions of this article will be made available by the authors, without undue reservation.
